# Synergetic Effect of Fullerene and Fullerenol/Carbon Nanotubes in Cellulose-Based Composites for Electromechanical and Thermoresistive Applications

**DOI:** 10.3390/polym17243259

**Published:** 2025-12-07

**Authors:** Ane Martín-Ayerdi, Timur Tropin, Nikola Peřinka, José Luis Vilas-Vilela, Pedro Costa, Vasil M. Garamus, Dmytro Soloviov, Viktor Petrenko, Senentxu Lanceros-Méndez

**Affiliations:** 1 BCMaterials, Basque Center for Materials, Applications and Nanostructures, UPV/EHU Science Park, 48940 Leioa, Spain; 2 European Molecular Biology Laboratory, 22607 Hamburg, Germany; 3 Innovative Macromolecular Materials Group (Imacromat), Physical Chemistry Department, Faculty of Science and Technology, University of the Basque Country UPV/EHU, 48940 Leioa, Spain; 4 Physics Centre of Minho and Porto Universities (CF-UM-UP), Laboratory of Physics for Materials and Emergent Technologies, LapMET, University of Minho, Campus of Gualtar, 4710-057 Braga, Portugal; 5 IB-S Institute of Science and Innovation for Sustainability, Universidade do Minho, 4710-057 Braga, Portugal; 6 Helmholtz-Zentrum Hereon, 21502 Geesthacht, Germany; 7 Ikerbasque, Basque Foundation for Science, 48009 Bilbao, Spain

**Keywords:** polymer nanocomposites, fullerene, piezoresistivity, thermoresistivity, hydroxypropyl cellulose, sustainability

## Abstract

A water-soluble hydroxypropyl cellulose (HPC) polymer matrix has been filled with different weight percentages (wt.%) of multiwalled carbon nanotubes (MWCNTs), fullerenes C_60_, fullerenols C_60_(OH)_24,_ and their combinations. We study the potential of the 0D nanoparticles for improving electrical properties of the conductive MWCNT network in a biocompatible matrix. Physicochemical effects of fillers content, both individually and in combinations (MWCNTs/C_60_ and MWCNTs/C_60_(OH)_24_), for these composite systems, have been investigated. The performed SAXS analysis shows improved nanofiller dispersion for films with two fillers. The electrical percolation threshold (P_c_) in MWCNTs composites occurs at ≈1.0 wt.%. A synergistic effect for binary filler composites on the electrical conductivity has been evaluated by keeping a constant amount of 0.5 wt.% MWCNTs (σ ≈ 3 × 10^−9^ S·m^−1^) and increasing the amount of C_60_ or C_60_(OH)_24_. A large increase in the electrical conductivity is obtained for the bifiller composites with 0.5 wt.% MWCNTs and 1.5 wt.% of C_60_(OH)_24_, reaching σ ≈ 0.008 S·m^−1^. Further, the sensing properties of 4.0/1.0 MWCNT/C_60_ nanocomposites were demonstrated by measuring both piezoresistive (PR) and thermoresistive (TR) responses. The combination of semiconductive fullerene/fullerenols combined with MWCNTs allows obtaining more homogeneous composites in comparison to single MWCNTs composites and also gives possibilities for tuning the electrical conductivity of the system. Overall, it is demonstrated that the use of bifillers with a water soluble biopolymeric matrix allows the development of eco-friendly high-performance electroactive materials for sustainable digitalization.

## 1. Introduction

The fast development of the Internet of Things (IoT) has boosted the design of new sensors to gather data from the surrounding environment [1]. However, the fast production and obsolescence of electrical and electronic equipment (EEE) have led to a growing problem of end-of-life (EoL) waste. No longer functional equipment contributes to growing electronic waste (E-Waste) being a major environmental concern [2]. In fact, E-Waste is composed of different materials, complicating their recyclability and reusability. Therefore, the proper selection of materials (i.e., renewable, recyclable or degradable) and processing methods for the production of electronic components and devices is essential on the path towards more sustainable electronics [3,4].

In this context, ongoing research efforts are focusing on the development of sustainable electronics by using composites-based environmentally friendly polymeric matrices, solvents and electrically conductive fillers [5,6,7]. Herein, biopolymers emerge as an alternative to petroleum-derived polymers due to their biodegradability or renewable characteristics [8]. In particular, cellulose is a natural resource obtained from plants, algae, and organisms [9]. Further, the environmental friendliness of a given polymer is also determined by the processing conditions, e.g., the liquid used in solvent-based additive manufacturing technologies, including screen-printing, direct ink writing or ink-jet printing [10,11]. Cellulose cannot be dissolved in water and is difficult to dissolve in organic solvents. Chemically modified cellulose derivatives, including carboxymethyl cellulose (CMC) or hydroxypropyl cellulose (HPC), offer the advantage of being water soluble, avoiding the use of hazardous organic solvents [12,13].

The addition of electrically conductive carbon nanofillers (e.g., carbon nanotubes, CNT; graphene oxide, GO or carbon black, CB) to non-conductive polymers is one of the strategies for obtaining electrically conductive composites [14]. In particular, CNTs are 1D cylindrical geometry materials, highly used due to their high aspect ratio, allowing the obtainment of electrically conductive composites at low filler concentrations [15]. Several studies have been reported regarding polymer/CNT composites for producing conducting paths, tracks and different sensor applications, including piezoresistive [16], or thermosensitive [17]. With the same purpose, composites have also been developed using 2D nanofillers, such as graphene (G) [18] and carbon nanohorns [19], as well as with carbon black. The latter, in comparison to 1D and 2D materials, requires addition of the highest amounts in order to enhance conductivity of the composite. The combinations of electrically conductive materials [20], most often CB with other nanofillers, for example, CNT/CB, have been shown to enhance the final properties of the materials [21]. Thus, the broad tailoring capabilities of bifiller carbon nanocomposites are being investigated and tested for various applications. Here, researchers mainly rely on CNT, GO and CB fillers and their combinations [22,23].

On the other hand, other 0D allotropic forms of carbon and their derivatives, including fullerene C_60_ and fullerenols C_60_(OH)_24_ have received less attention. These quasispherical carbon nanoparticles with a typical size of about 1 nm, and their derivatives, present physicochemical properties that have been explored for applications in the fields of photovoltaics [24], or biomedicine [25]. Composite-wise, the research on modifications of properties of polystyrene on addition of C_60_ can be mentioned [26]. One recent papers using fullerenes implies positioning of fullerenes mixed with nanotubes and graphene on top of Styrene-b-(ethylene-co-butylene)-b-styrene (SEBS) thin film during preparation [27]. The highest stretchability of SEBS is then combined with electrical conductivity for sensing applications.

In this research, we investigate the possibility of combining multiwalled carbon nanotubes (MWCNTs), as conductive carbon nanofillers, with fullerenes to tune the electrical properties and the sensing characteristics. Herein, water-soluble HPC has been selected as the polymer matrix, water as the solvent, and MWCNTs, fullerenes and fullerenols as fillers.

Typically, fullerenes are well dispersed in nonpolar solvents while specific synthesis procedures are necessary to obtain fullerenes aqueous solutions [28]. In order to maintain a sustainable approach, the use of typical organic fullerene solvents has been avoided. Also, to evaluate processability and composites characteristics achieved by using water dispersions of pristine fullerenes, the functionalized fullerene C_60_, fullerenols C_60_(OH)_24_ have also been used. The added hydroxyl groups allow fullerenols to be dispersed in water, enhancing applications in green chemistry [29,30]. Following the study of the physicochemical properties of single fillers composites (HPC/MWCNT, HPC/C_60_, and HPC/C_60_(OH)_24_), binary filler nanocomposites (HPC/MWCNT/C_60_ and HPC/MWCNT/C_60_(OH)_24_) have been prepared and investigated. The electromechanical and thermoresistive responses of selected materials were evaluated as suitable functional materials for sensing applications.

## 2. Materials and Methods

### 2.1. Materials

Hydroxypropyl cellulose (HPC), average M_w_ of ~100.000 g mol^−1^ (191884-100G), has been purchased from Sigma Aldrich. Fullerene (C_60_) (TCI, purity > 99.0%), fullerenols (C_60_(OH)_22-24_, MST-Nano EU, Riga, Latvia) and multi-walled carbon nanotubes (MWCNTs, NC7000™, Nanocyl, SA, Sambreville, Belgium, purity ~90%, diameter of <*d*>~9.5 nm, length of <*L*>~1.5 µm) were used as nanofillers. Finally, distilled water has been used as solvent.

### 2.2. Nanocomposite Preparation

Thin films of pure polymer (blank) and the corresponding nanocomposites have been prepared by the solution casting method. Initial HPC biopolymer solutions have been obtained by dissolving 10% weight/volume (*w*/*v*) HPC in ultrapure water following the procedure reported in [31]. For the further mixtures with nanoparticles, the methods implied maintaining HPC contents at 10% *w*/*v*. Three different procedures have been followed in the preparation of single and bifiller composites. The amount of filler included was based on the processability and the aimed electrical functional properties. The nomenclature that has been used and weight percentage (wt.%) of the filler added in the processing of single filler composites are displayed in Table 1 while for bifillers they are shown in Table 2. The same samples have been used in all the characterization procedures.

In the first procedure, HPC/C_60_ composites have been prepared from solutions obtained by dispersing different wt.% of fullerene in water via magnetic stirring for 1 h at room temperature (RT). Then, HPC polymer in the previously mentioned *w*/*v* % was added, and the solution which was magnetically stirred at RT until the polymer was totally dissolved. Solutions for HPC/C_60_(OH)_24_ composites have been prepared following the same procedure.

During preparation of HPC/MWCNTs composites, HPC was first dissolved in water using magnetic stirring at RT. In order to disperse the MWCNTs, they were placed in a separate vial with water. Then, this solution was ultrasonicated for 3 h at a controlled temperature between 25 and 30 °C before being mixed with the polymer solution. To reach homogeneous mixing, the solution was next magnetically stirred for 1 h more at RT.

During preparation of composites with mixtures of two fillers, a combination of the previous methods was used. First, HPC/C_60_ and HPC/C_60_(OH)_24_ solutions were obtained following the respective first procedure. Then, MWCNTs were ultrasonicated in water, following the second procedure, and added to the prepared HPC/C_60_ and HPC/C_60_(OH)_24_ solutions. The mixed solution was magnetically stirred for 1 h at RT.

To choose the filler contents for bifiller composites, two different approaches were followed. First, the amount of MWCNTs was fixed at 0.5 wt.% while the wt.% of fullerene and fullerenols was increased. As it will be presented further, the initial composite (low contents or no C_60_) was not conductive, while the ones with higher C_60_ wt.% were above the percolation threshold. Also, MWCNTs/C_60_ and MWCNTs/C_60_(OH)_24_ samples were prepared with an overall higher filler content. The amount of filler included (4 wt.% of MWCNTs) was selected after the investigation of the electrical properties of the composites.

In the final step of all three aforementioned procedures, nanocomposite films were prepared by doctor blade and dried overnight at RT in order to assure solvent evaporation. After one night, films with a thickness in the range of ~80–120 μm were obtained.

**Figure 1:**
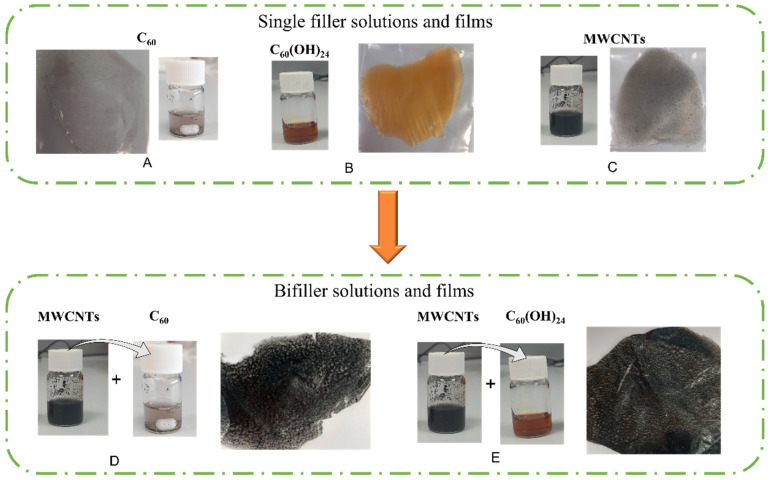
Solution and films morphology obtained after preparation process for (**A**–**C**)—single filler and (**D**,**E**)—bifiller composites.

The final solutions and films morphology after drying are demonstrated in Figure 1. Solution and films for single filler composites develop brown/white colour for C_60_ (A), an orange colour for C_60_(OH)_24_ (B) and black colour for MWCNTs (C). For the case of bifillers, mostly black coloured films were obtained. As it can be observed, due to hydrophobic nature of fullerenes (D), multiple microaggregates are observed along the polymer matrix, whereas fullerenol based (E) films display the typical colour of the solution, indicating a well dispersed filler. In order to make a comparison, films on Figure 1 are presented for different filler contents (smaller for A–C, higher for D,E).

### 2.3. Physicochemical Characterization

The structure and physicochemical properties of nanocomposite thin films have been studied using several methods. Composite morphology was evaluated by Scanning Electron Microscopy (SEM). The SEM images were obtained using a Hitachi S-4800 set up at an accelerating voltage of 5 kV with magnification of 20,000×.

Infrared spectra were collected with a Nicolet Nexus Fourier transform infrared spectrophotometer (FTIR, Thermo Fisher Scientific, MA, USA). The FTIR spectra were obtained in the attenuated total reflection (ATR) mode in the range from 400 to 4000 cm^−1^ at a resolution of 4 cm^−1^ and 64 scans per spectrum. This characterization was used to assess the possible interactions between the nanofillers and polymer.

Small-angle X-rays scattering (SAXS) was used to investigate organization of nanofillers in the matrix. The measurements were performed at the P12 BioSAXS Beamline at PETRA III ring (EMBL/DESY) in Germany [32]. The sample-to-detector distance, 3.1 m, gave the q-range of 0.05–5.9 nm^−1^, calibrated using silver behenate [33]. Scattering patterns were obtained with a Pilatus 6 M pixel detector. The scattering by samples in form of thin films was measured at 9 different points, distanced by 0.3 mm, forming a square with a point in the middle. For each point, ten consecutive frames (0.1 s each) measurements were performed. All scattering curves of a recorded dataset were compared to a reference measurement (typically the first exposure) and finally integrated by an automated acquisition programme. This approach allowed us to verify the absence of artefacts due to radiation damage. The scattering from pure films was measured as well. All the resulting data were normalized to the transmitted beam.

Differential scanning calorimetry (DSC) was performed using a Waters^TM^ TA instruments model DSC 25. Samples with a mass of ~10 mg, sealed in aluminum pans, were subjected to a heating scan from −70 to 200 °C at a scanning rate of 10 °C·min^−1^ under constant nitrogen flow (20 mL·min^−1^). Additionally, some samples were analyzed by heating/cooling/heating scans from −70 to 200 °C, with a scanning rate of 10 °C·min^−1^ under constant nitrogen flow (20 mL·min^−1^).

Mechanical properties measurements were carried out in the tensile mode, using a universal testing machine Shimadzu, model AG-IS, with a load cell of 500 N. Rectangular samples with dimensions of 10 × 20 mm^2^ and thickness in the range from 80 to 120 μm were prepared and measured at room temperature at a test velocity of 1 mm·min^−1^. The elastic modulus was calculated at 0.5% of strain. Three samples extracted from the same composite film were measured consecutively.

The electrical conductivity was evaluated by measuring current–voltage (I–V) characteristics in DC mode using a Keithley 487 picoammeter/voltage source, with the applied voltage ranging from −4 V to +4 V at room temperature. Electrical conductivity (σ) was calculated using the following equation:

(1) $$\sigma =\;\frac{1}{R}\;\times \;\frac{l}{A}$$

Prior to the measurements, gold electrodes with a diameter of 5 mm were deposited via magnetron sputtering (Quorum Q150T S) on both sides of the film in a parallel configuration. At least three measurements within the same film (at the far-standing points) were performed.

### 2.4. Functional Characterization

Piezoresistive and thermoresistive measurements were performed in surface mode after placing conductive silver (AGG3790, Agar Scientific, Rotherham, UK) electrodes at the surface of the films with about 10 mm length and at a distance of 10 mm between them. Piezoresistive measurements were performed in 4-point-bending mode at a deformation rate of 1 mm·min^−1^ up to a maximum deformation of 5 mm. With respect to the stability test of the sensing response, 160 consecutive loading-unloading cycles were evaluated up to a bending of 1 mm at 5 mm/min rate. The electrodes were placed in the bottom surface of the samples, and the electrical resistance (digital multimeter Agilent 34401A) variation was measured under deformation in real time. The piezoresistive sensitivity was evaluated through Equations (2) and (3) [34]:

(2) $$GF=\;\frac{∆R/R_{0}}{\varepsilon },$$

(3) ε=35zta2,

where ∆R is the electrical resistance variation, $R_{0}$ is the electrical resistance in the undeformed samples, ε is the applied strain, *t* the thickness of the samples, z is the deformation along the zz axis and *a* is the distance between bending points [34].

The thermoresistive sensitivity (S) measured in surface mode was obtained by varying the temperature (ΔT) between 30 and 50 °C at a rate of 5 °C·m^−1^ in a Linkam THMSE 600, while simultaneously measuring the electrical resistance variation. The following expression was used:

(4) $$S=\;\frac{∆R/R_{0}}{\Delta T}.$$

## 3. Results and Discussion

### 3.1. Morphological and Chemical Analysis

**Figure 2:**
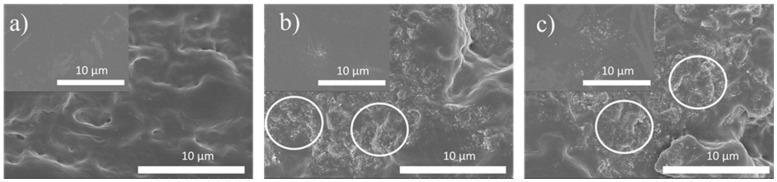
Representative SEM images of (**a**) pristine HPC, (**b**) 4.0/1.0C_60_ and (**c**) 4.0/1.0.C_60_(OH)_24_ nanocomposites. Main images are the films cross-sections, insets—film surfaces.

The morphology of the composites was evaluated with the help of SEM images of films cross-sections (Figure 2), and their surfaces (Figure 2, insets). The HPC matrix (Figure 2a) has a compact structure with no visible pores or voids. The distribution of filler is shown for the 4.0/1.0 content composites, as representative of the rest of the samples. Figure 2b,c, in particular in the cross-section scans, show well distributed small MWCNT agglomerates (as bright spots) within the polymer matrix [35]. On the other hand, fullerenes and fullerenols, cannot be seen in these images, as the sizes of these nanofillers (~1–5 nm) are below resolution.

**Figure 3:**
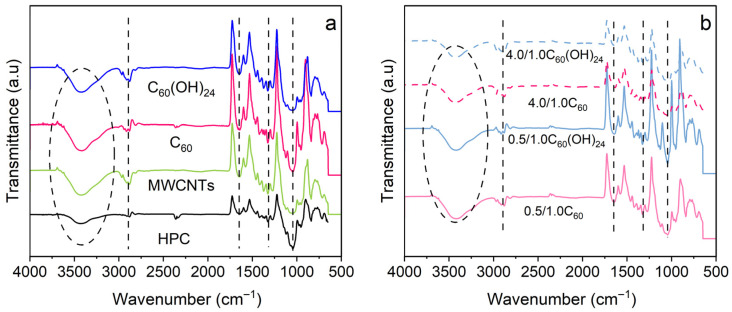
ATR-FTIR spectra of neat biopolymer and composites. Identified peaks have been highlighted with the dashed lines and an ellipse. (**a**) Single filler composites spectra with corresponding 1.5 wt.% filler content and (**b**) binary composites with the nanofiller contents as designated under each spectrum.

Possible interactions between the nanofillers and the matrix were analyzed by Fourier transform infrared (FTIR) spectroscopy (Figure 3). For pristine HPC, the main peaks are marked with dashed lines: 3610–3307 cm^−1^ (O-H stretching of hydroxyl groups), 2972 cm^−1^ (C-H stretching), 1652 cm^−1^ (O-H bending), 1376 cm^−1^ (CH_2_ bending) and 1056 cm^−1^ (C-OH stretching) [31]. The spectra for samples containing 1.5 MWCNTs, 1.5 C_60_ and 1.5 C_60_(OH)_24_ for single fillers (Figure 3a) and 0.5/1.0C_60_ and 0.5/1.0C_60_(OH)_24_ for bifiller (Figure 3b) composites are also shown. No peaks displacement or new peaks in the composite spectra compared with the HPC spectra are revealed. In addition, on Figure 3b, the spectra of 4.0/1.0C_60_ and 4.0/1.0C_60_(OH)_24_ are included. The results are representative for the rest of the samples and confirm that there are no specific chemical interactions between nanofillers and HPC. Nevertheless, the inclusion of filler significantly affects the physicochemical properties as it will be described further.

### 3.2. Small-Angle X-Ray Scattering

**Figure 4:**
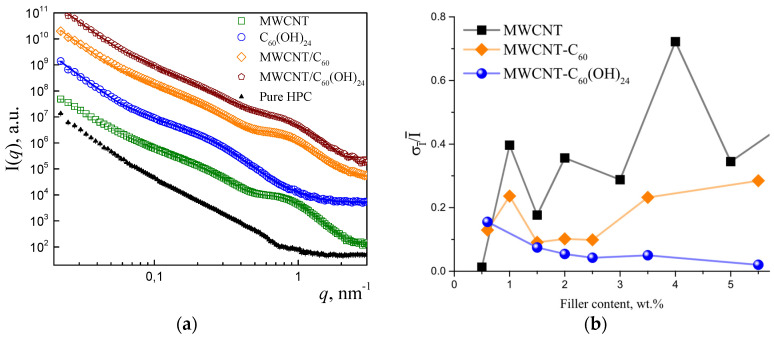
(**a**) SAXS experimental data (symbols) and fits (straight lines) for HPC nanocomposite thin films, containing 1.0MWCNT, 1.5fullerenols, and 4.0/1.0C_60_ and 4.0/1.0C_60_(OH)_24_ bidisperse fillers—MWCNT/C_60_ and MWCNTs/C_60_(OH)_24_. Curves shifted along *y*-axis by consecutive multiplication. (**b**) Characteristic dependence of films and fillers dispersion quality in the thin films after several measurements of the same sample ($\sigma_{\bar{I}}/\bar{I}$) according to SAXS data. Three different HPC nanocomposites are compared at different weight percentages. MWCNTs have been measured for 0.5 to 5.0 wt.% contents while in the case of fillers, the 0.5 wt% MWCNTs was maintained, the amount of the second filler was increased. The filler wt.% represent the sum of both fillers’ contents.

The nanostructure and dispersion state of the carbon nanofillers in the nanocomposite thin films have been characterized by SAXS. Despite water not being the most effective solvent for carbon nanofillers (agglomeration observed), it was intentionally used in combination with the water-soluble HPC matrix to proceed with the green and sustainable processing principles, as required for environmentally friendly printed electronics [36]. The characteristic curves are presented in Figure 4a for different films with single fillers in the matrix, and for the composites incorporating two fillers as well. For all the measurements, the low-q scattering is dominated by the micro inhomogeneities in the biopolymer film. This respective scattering has been incorporated in the fitting models via two power-law function:

(5) $$I\left(q\right)=\left\{\begin{array}{c} Aq^{-p_{1}},\;\;\;q\leq q_{c}\\ Bq^{-p_{2}},\;\;\;q>q_{c} \end{array}\right.,$$

where *p*_1_ and *p*_2_ are the power-law exponents and *A* and *B* are the scale parameters, related as $B=Aq_{c}^{p_{2}}/q_{c}^{p_{1}}$.

The scattering by the nanofillers is distinguished in the *q*-range of ~0.1–2 nm^−1^ (which corresponds to sizes ~2$\frac{\pi }{q}=60-3\;\mathrm{nm}$). For some of the nanofillers we observe qualitative differences in SAXS curves. The samples containing nanotubes are characterized by a broad peak around *q*~0.8 nm^−1^. On the other hand, fullerenols are characterized by a weaker broad peak at *q*~0.2 nm^−1^, which can be distinguished from MWCNTs. To gain structural information on the samples in more details, the curves have been fitted by the respective analytical expressions [37].

For the nanotubes, similar to previous results in the literature [38], the SAXS curves can be fitted well by the core–shell cylinder model with size polydispersity. The form-factor of a core–shell cylinder is:

(6) $$P_{CS\;cyl}\left(q\right)={\left(V_{1}{∆\rho }_{1}\frac{\sin\frac{q(L+2T)\cos\theta }{2}}{\frac{q(L+2T)\cos\theta }{2}}\frac{2J_{1}\left(qR_{1}\sin\theta \right)}{qR_{1}\sin\theta }+V_{c}{∆\rho }_{c}\frac{\sin\frac{qL\cos\theta }{2}}{\frac{qL\cos\theta }{2}}\frac{2J_{1}\left(qR_{c}\sin\theta \right)}{qR_{c}\sin\theta }\right)}^{2},$$

where *L* is the cylinders length, *T*—shell thickness, *R*_c_, *V*_c_—the respective radius and volume of cylinders core, *R*_1_ = *R*_c_ + *T*, and *V*_1_—whole cylinders volume, Δ*ρ* is the corresponding contrast, *J*_1_—the first order Bessel function. This expression was averaged over angle θ and the distribution of cylinders radii. The obtained internal radius and shell thickness are roughly 2.5 nm each, which results in a total diameter of ~10 nm in accordance with the value reported by the manufacturer. This result indicates a good dispersion of the MWCNTs in the film. An estimate of the average lengths of the nanotubes straight segments cannot be made from the data due to growing impact of scattering by the matrix at *q* < 0.5 nm^−1^. A bend in the curve, that can be related to the *q*^−1^ scattering by the elongated objects is observed at *q*~0.1 nm^−1^. Yet, the values of respective *q*_min_, using which the cylinder length *L* can be estimated as π/*q*_min_, cannot be reliably extracted.

The scattering by fullerenes/fullerenols was modelled as of a system of polydisperse homogeneous balls. The respective form-factor is given by:

(7) $$P_{sph}\left(q\right)={\left(\frac{3}{V}\frac{\sin qR-qR\cos qR}{{\left(qR\right)}^{3}}\right)}^{2},$$

where *R* is the radius. The fullerenol clusters in the film present an average radius <*R*>~5 nm. For comparison, in reference [39] the specifically synthesized fullerenols C_60_(OH)_12_ developed a narrow size-distribution with lower sizes (~2 nm) at 0.1 wt.%. The larger size of particles in the nanocomposite in the present case is due to a higher concentration, as well as different media (polymer matrix vs. water solution) [40]. For the samples containing pristine fullerenes we did not observe scattering corresponding to <100 nm particles, as expected for the water solutions of these hydrophobic particles [41]. Finally, for those nanocomposites containing mixtures of fillers, the fits have been performed using sums of the corresponding scattering functions.

Additional analysis of scattering by films at different regions of the surface allows for the testing of films homogeneity at the microscale. As described in the Methods section (Section 2), all films were measured at nine different points. For some films, differences in *I*(*q*) between these points were observed, reflecting variation in films thickness and fillers dispersion. To evaluate this, for each point the average SAXS intensity $\bar{I}$ was calculated in the *q* range of 0.1–2 nm^−1^. Afterwards, the two values most apart from the median value were discarded and the respective mean average displacement was calculated. The dependence of this value on filler content for different sample series is plotted on Figure 4b.

The dispersion quality (σ_i_/I) of single MWCNTs have been scanned from 0.5 to 5.0 wt.% The results of MWCNTs have been used as a reference with the aim of analyzing dispersion effect with the incorporation of C_60_ and C_60_(OH)_24_. For the bifillers, the concentration of MWCNTs has been set at 0.5 wt.% while increasing the amount of the second filler. It can be observed that films inhomogeneity increases for the nanocomposites containing MWCNTs as single fillers, decreases for bifiller MWCNT/C_60_(OH)_24_ films, and behaves non-monotonically for MWCNT/C_60_. For the bidispersed fillers the improvement of films structural quality is observed for 1–2 wt.% of added fullerenes or fullerenols.

### 3.3. Thermal Properties

**Figure 5:**
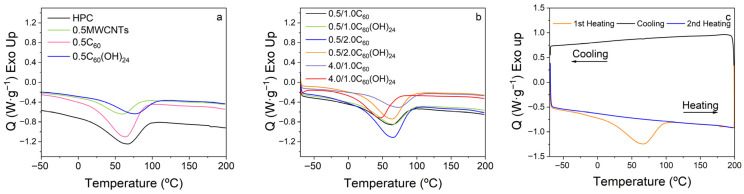
Representative DSC thermograms: (**a**) pristine polymer and single filler composites; (**b**) mixed bifiller composites containing fullerene and fullerenol; (**c**) heating/cooling/heating cycle (pure HPC).

The thermal properties of the films have been evaluated by the DSC, respective thermograms are presented in Figure 5. For all the cases, pure/single (Figure 5a) and for two fillers (Figure 5b), endothermic peaks were observed at the temperature about 60 °C with minor changes from film to film. These peaks are related to the water absorbed by the cellulose films [42,43]. To clarify, a heating/cooling/heating cycle has been performed for the pure HPC film (Figure 5c, each scan shown with different colour). While during the first heating (black), the endothermic peak was observed, during the second heating (blue) the peak was no longer present.

### 3.4. Mechanical Properties

**Figure 6:**
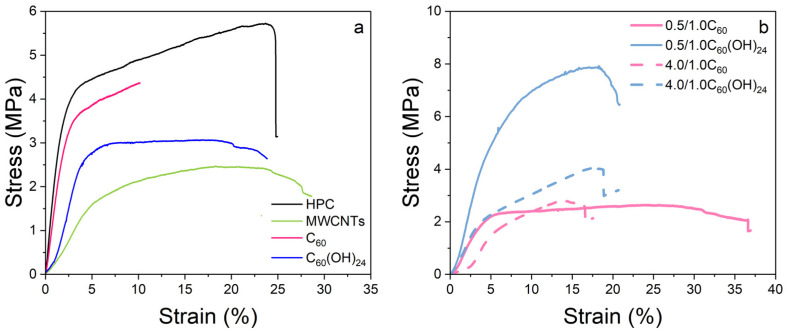
Stress–strain curves of (**a**) pristine polymer, HPC, and single filler of different type at 0.5 wt.% and (**b**) composites containing a mixture of two nanofillers.

The stress–strain tests of the samples were carried out in the tensile mode with the objective of analyzing their behaviour for posterior piezoresistive applications. Figure 6 shows some representative stress–strain curves for pristine HPC and the respective composites films for both single fillers and bifillers cases. Mechanical properties of HPC were compared with the composites. The effect of adding a single filler or two fillers was checked, as well as the possible mechanical changes due to different weight percentage of filler incorporated into the composite. The prepared HPC films display a maximum strain (ԑ) of ԑ ≈ 25 ± 7% and a Young modulus of E ≈ 240 ± 30 MPa.

In Figure 6a, a comparison between different single fillers at 0.5 wt.% content is presented. The choice of this concentration is related to the electrical properties of composites, as explained in the next section. Similar strain-at-rupture values to that of the polymer matrix were obtained for MWCNTs and C_60_(OH)_24_ composites, while the C_60_ composites exhibited lower strain to rupture. With respect to the elastic zone, C_60_ composites present comparable behaviour, with an elastic modulus *E* ≈ 180 ± 50 MPa. Conversely, for MWCNTs and C_60_(OH)_24_ nanofillers, the composite reduces its mechanical properties as compared to the polymer matrix.

Mechanical properties of bifiller composites have been analyzed for both 0.5 wt% MWCNTs-containing films, and a film with high nanofiller content (see Table 2). For both cases, lower strain values in comparison to the single filler samples have been observed, with the exception of 0.5/1.0C_60_ (demonstrated on Figure 6b). In the elastic zone, similar stress–strain curves have been obtained. In general, results reveal that the inclusion of two fillers leads to lower maximum strain values than those containing a single filler. Between fullerene and fullerenols, fullerene allows higher maximum strains, with the similar elastic zone. For the bifiller composites containing higher content of MWCNTs than fullerene/fullerenol (4.0/1.0 C_60_ and 4.0/1.0 C_60_(OH)_24_), the previously mentioned tendency of reduced maximum strain results is maintained. In contrast to previous results, the inclusion of fullerene allows a wider elastic zone range than with fullerenols.

### 3.5. Electrical Conductivity Properties

**Figure 7:**
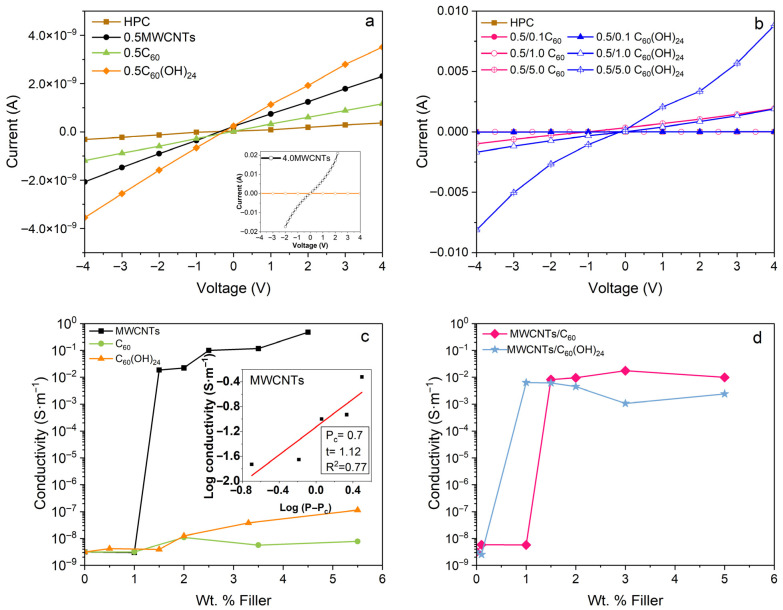
(**a**) Representative I-V curves at highest and lowest concentrations of C_60_, C_60_(OH)_24_ and MWCNTs composites. Inset: magnification for the composites above the percolation threshold. (**b**) Electrical conductivity of nanocomposites films containing 0.5MWCNTs and varying the wt.% of C_60_ and C_60_(OH)_24_. (**c**) Electrical conductivity as a function of filler content for the single filler composites. Inset: linear fit for obtaining P_c_ and t values for MWCNTs composites. (**d**) Electrical conductivity, maintaining constant 0.5MWCNTs, as a function of C_60_ and C_60_(OH)_24_ filler content for the bifiller composites.

The DC conductivity of the films was measured from the I–V curves presented in Figure 7a (single filler nanocomposites) and Figure 7b (bifiller systems). The respective σ values have been obtained using Equation (1). It is worth noting that, in Figure 7a, for the fullerene and fullerenol, I–V show a perfect linear Ohmic response, while the MWCNTs-containing films are characterized by a slightly non-lineal response, attributed to interfacial polymer-filler contributions [44]. In Figure 7b a non-linear behaviour is observed as well, owing to the addition of MWCNTs to C_60_ and C_60_(OH)_24_.

Electrical conductivity dependencies (Figure 7c) for the composites containing only MWCNTs as fillers show an increase in conductivity from ~3 × 10^−9^ S·m^−1^ for 0.5MWCNTs to ~0.02 S·m^−1^ for 1.0MWCNTs. This change reflects that the percolation threshold, P_c_, has been reached and passed. The value of P_c_ is calculated using:

(8) $$\sigma ={\sigma_{0}(P-P_{c})}^{t}$$

where P is filler concentration in the matrix, σ_0_ is the electrical conductivity and *t* is a critical exponent related to the dimension of the conductive network [45]. It is known that the high aspect ratio of MWCNTs allows for the obtainment of lower values of P_c_. The literature reports P_c_ values for different polymeric matrices, depending on the distribution of the filler and the matrix. SEBS combined with CNTs presented a P_c_ in the range between 1 and 2 wt.% [46] whereas cellulose composites with single walled carbon nanotubes (SWCNTs) show a P_c_ around 2.0 wt.% [47]. In the present case, a P_c_ of ~0.7 wt.% MWCNTs, t exponent value of 1.12, with R^2^ 0.77 (Figure 7c inset) have been obtained.

Furthermore, the maximum electrical conductivity value, 0.48 S·m^−1^ was obtained for the 4.0 wt.% MWCNTs content composite (Figure 7c). In the case of C_60_ and C_60_(OH)_24_ no significant conductivity variation (7.78 × 10^−9^ S·m^−1^ and 1.15 × 10^−7^ S·m^−1^, respectively) has been observed on increasing filler content up to 5.0 wt.%, consistent with the insulating character of these fillers. Similarly, a slight increase in conductivity with respect to the polymer, has been reported for poly (phenylene sulphide) (PPS) and polyethylene oxide (PO)/fullerene (C_60_) composites [48,49].

Synergetic effect between carbonaceous electrically conductive fillers has been reported to modify the P_c_ value compared to the single filler composites [50,51]. As a relevant example, epoxy resin matrix composites show a P_c_ at 0.2 wt.% MWCNTs filler content and 15 wt.% for graphene-like nanoplatelets (GNP), and 35 wt.% for graphite. The synergetic effect of these fillers was studied with the combination of CNTs at 0.1 wt.% and different amounts of added GNP or graphene. The results revealed that P_c_ was achieved at 1 wt.% of GNPs and 2 wt.% graphite for the 0.1 wt.% CNTs containing composite [52]. Additionally, a polystyrene (PS) matrix was filled with CNTs and CB nanoparticles. CNTs-filled nanocomposites had a P_c_ at 0.25 wt.%, while for CB P_c_ was 2.0 wt.%. The combination of both fillers at 75(CNTs):25(CB) ratio exhibited lower resistance than individual CNTs containing composites. Thus, a synergetic effect was observed in this system [53].

In the present work, the effect of fullerenes addition to MWCNTs containing HPC nanocomposites was studied (Figure 7d, pink line). As it was previously shown, the HPC/MWCNTs composites are not conductive at 0.5 wt.% filler content (σ≈ 3.0 × 10^−9^ S·m^−1^). For the study of MWCNT-fullerene synergistic effects, the 0.5CNT sample was used as the main/reference matrix, with different wt.% of C_60_ and C_60_(OH)_24_ then added. Measurements reveal that by adding increasing amounts of C_60_ to the 0.5MWCNTs system, a strong increase in the electrical conductivity by several orders of magnitude was observed at 1.5C_60_ content. Similarly, by adding fullerenols (Figure 7d, blue line), the percolation was reached at 1.0C_60_(OH)_24_. For both fillers we observed the typical percolation-like electrical conductivity curve. Similarly to these results, improved electrical conductivity has been reported for C_60_ in the polydivinylbenzene (PDVB) polymer coated with polyaniline (PANI) intrinsically electrically conductive polymers. Reported results showed an electrical conductivity of 7.9 × 10^−10^ S·m^−1^ for the polymer, whereas PANI/C_60_ bifiller composites showed an increased conductivity of 10 orders of magnitude compared to the polymer [54].

After the analysis of bifillers synergetic effect, a selection of composites was conducted for functional characterization. The piezoresistive behaviour resulted from a balance between the composite’s electrical resistance and its response to applied mechanical stress. For the mechanical properties, the inclusion of filler highly affects the mechanical properties (see Figure 6b) of the composites. At the same time, electrical conductivity values measured at the plateau region correspond to a stable conductive network, providing a reproducible piezoresistive signal.

In this sense, 4.0MWCNTs sample (Figure 7c) was selected for functional characterization owing to its high electrical conductivity. In order to analyze the effect of the second filler, 1.0 of C_60_ and C_60_(OH)_24_ was added to 4.0MWCNTs sample. The choice of 1.0 wt.% was based on observed mechanical properties.

**Figure 8:**
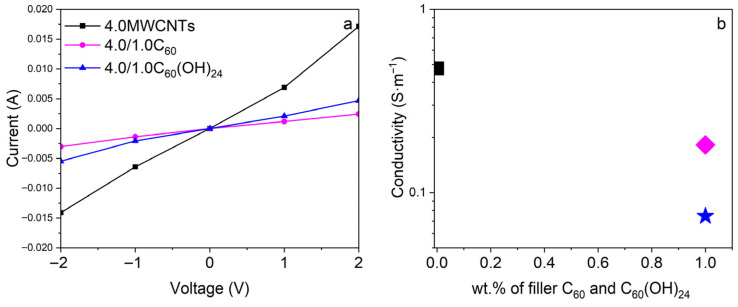
(**a**) I–V curves for bifiller composites, with the same amount of MWCNTs, 4 wt.%. (**b**) electrical conductivity values for the single 4.0MWCNTs (black) and bifillers 4.0/1.0C_60_ (pink) and 4.0/1.0C_60_(OH)_24_ (blue).

In Figure 8a, I–V curves of single and bifiller composites are shown and the corresponding electrical conductivities are presented in Figure 8b. The electrical response is the one expected as a consequence of previous measurements, where single filler possesses higher conductivity than bifillers. In addition, fullerenes show higher electrical conductivity than fullerenols.

### 3.6. Piezoresistive and Thermoresistive Properties

Finally, the biopolymer-based composites have been evaluated in terms of their piezoresistive (PR) and thermoresistive (TR) capabilities exhibiting a change in electrical resistance under mechanical stress and temperature variation, respectively. The respective performance of the selected composites for resistance-strain tests was quantified by the Gauge factor (GF) whereas for resistance-temperature tests, it was quantified by the thermoresistive sensitivity (S).

The sensing ability is strongly affected by filler type and content and filler/polymer interactions [55]. The largest active response is typically found above the P_c_ based on the larger electrical conductivity and larger variation in the percolation network, e.g., under mechanical solicitation.

**Figure 9:**
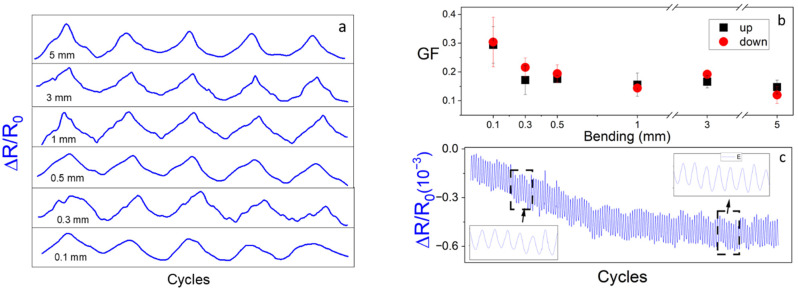
(**a**) Piezoresistive response of the 4.0/1.0C_60_ composite in 4-point bending at different maximum deformations from 0.1 to 5 mm. (**b**) Piezoresistive sensitivity of the composites as a function from 0.1 to 5 mm and (**c**) relative resistance variation in bending mode during 160 cycles for 1 mm of bending.

Figure 9a shows the relative electrical resistance ($∆R/R_{0})$ variation under bending for the 4.0/1.0C_60_ composite with maximum bending deformation from 0.1 to 5 mm during five cycles. The electrical resistance increases upon bending and for all the tests the electrical resistance was returned to the initial value when the stimulus was released, indicating the reversibility of the process and the recovery of the initial conductive network configuration. The piezoresistive sensitivity variations are characterized by a GF between 0.13 and 0.3, nearly independent of the maximum bending deformation, as shown in Figure 9b. Thus, even while the GF is not particularly high, it is enough to enable sensing applications. To test the material’s stability under repeated cyclic bending, tests up to 160 cycles were further carried out (Figure 9c). There is an overall decrease in the maximum and minimum resistance over cycling, due to irreversible reconfigurations of the conductive network and/or stress relaxation of the polymer matrix, that tends to stabilize after 60 cycles. Nonetheless, the R_min_–R_max_ range remained constant along the cycles and so does the GF sensitivity. The demonstrated performance of the composite showed that the biopolymer with carbon fillers can be an excellent alternative to the petroleum-based composites for piezoresistive applications.

**Figure 10:**
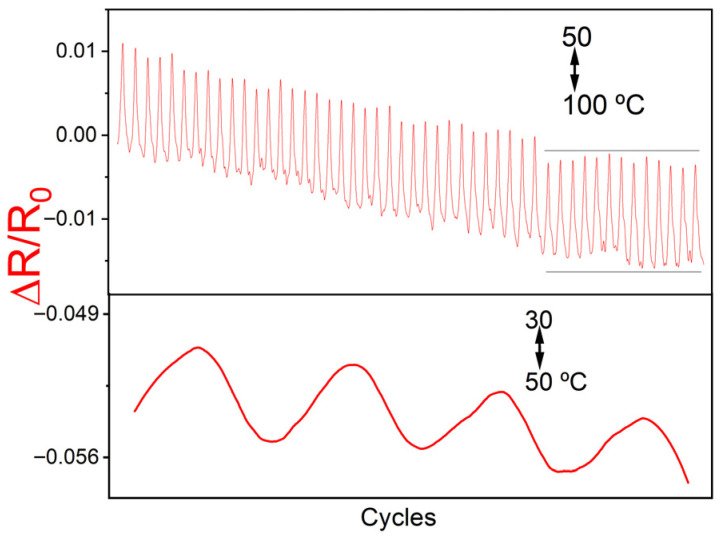
Thermoresistive response of composites with 4.0/1.0C_60_ filler content for several cycles, in the temperature range 50–100 °C (**top**), and 30–50 °C range (**bottom**).

The thermoresistive response of the 4.0/1.0C_60_ composite was also studied (Figure 10). An increase in electrical resistance with increasing temperature and a decrease with decreasing temperature were observed, reflecting a positive thermoresistive coefficient. A slight decrease in the minimum and maximum resistance during the first cycles was also observed, due to initial irreversible effects. Nonetheless, the response was stabilized after approximately 30 cycles. The thermoresistive response is $S_{30-50}$ = (1.6 ± 0.2) × 10^−4^ °C^−1^ and $S_{50-100}$ = (2.4 ± 0.3) × 10^−4^ °C^−1^, is also suitable for sensing applications. Other composites with similar electrical conductivity present similar thermoresistive behaviour and sensitivity.

Thus, piezoresistive and thermoresistive sensitivities were achieved for the conductive composites combining MWCNTs and fullerene nanofillers. Even though slightly lower functional properties than those reported on cellulose-based composites were obtained [56,57], the use of sustainable biopolymer-based composites with carbonaceous fillers and processing methods demonstrates them here as sustainable materials with multifunctional thermo/piezoresistive properties.

## 4. Conclusions

The combination of water soluble HPC matrix and MWCNTs/C_60_, MWCNTs/C_60_(OH)_24_ nanofillers was explored for the development of environmentally friendly biopolymer-based sensors for sustainable electronics. A structural analysis of fillers dispersion in the matrix was performed using SAXS. The results show that bifiller composites form a more homogeneous distributed system than single MWCNTs composites, especially in the 1–2 wt.% range. When comparing the type of the second filler added to MWCNTs, C_60_ composites behaved more non-monotonically while the C_60_(OH)_24_ composites maintained the dispersion quality. The spectroscopical analysis that was performed revealed no filler-matrix interaction. Furthermore, the electrical properties of single filler and bifiller composites were analyzed. For MWCNT as a single filler MWCNTs, a percolation threshold at P_c_~0.7 wt.% and conductivity near σ ≈ 0.02 S·m^−1^ (for 1.0 wt.%MWCNTs) was obtained; while for fullerenes/fullerenol, only a slight increase in conductivity of the matrix up to 5 wt.% was observed. Electrical conductivity measurements show that the inclusion of fullerene/fullerenol allows tuning of the percolation threshold of composites. In the case of bifiller composites, a synergetic effect was found, with a strong increase in the electrical conductivity for the 0.5/1.5C_60_ and 0.5/1.0C_60_(OH)_24_ samples. Nanocomposite samples with higher fillers content, 4.0/1.0C_60_, show a piezoresistive response characterized by a GF between 0.13 and 0.3 and a thermoresistive response with a sensitivity of $S_{30-50}$ = 1.6 ± 0.2 × 10^−4^ °C^−1^. The nature of this effect can be further analyzed, for example, by mixing 0D with 1D carbon nanofillers in different matrixes, e.g., in thermoplastics like polystyrene or polyvinylidene fluoride.

The incorporation of C_60_ or C_60_(OH)_24_ as a second filler to MWCNTs composites provides one new perspective for electronic applications. The incorporation of fullerenols permits better fillers dispersion in water solvents, while as it seems, fullerenes already enable functionality. The synergetic behaviour observed for both nanoparticles and, together with the highlighted use of water-soluble biopolymer HPC matrix demonstrates the processability of functional composites.

## Figures and Tables

**Table 1:** Different wt.% of fillers added during preparation of single filler containing composites.

Sample	Wt.% C_60_	Sample	wt.% C_60_(OH)_24_	Sample	wt.% MWCNTs
0.5C_60_	0.5	0.5C_60_(OH)_24_	0.5	0.5MWCNTs	0.5
1.0C_60_	1.0	1.0C_60_(OH)_24_	1.0	1.0MWCNTs	1.0
1.5C_60_	1.5	1.5C_60_(OH)_24_	1.5	1.5MWCNTs	1.5
3.0C_60_	3.0	3.0C_60_(OH)_24_	3.0	3.0MWCNTs	3.0
-	-	-	-	4.0MWCNTs	4.0
5.0C_60_	5.0	5.0C_60_(OH)_24_	5.0	5.0MWCNTs	5.0

**Table 2:** Nomenclature and wt.% used for preparation of bifiller composites. The first numbers are the wt.% of MWCNTs, the second—wt.% of C_60_ and C_60_(OH)_24_.

Sample	wt.% MWCNTs/C_60_	Sample	wt.% MWCNTs/C_60_(OH)_24_
0.5/0.1C_60_	0.5/0.1	0.5/0.1C_60_(OH)_24_	0.5/0.1
0.5/1.0C_60_	0.5/1.0	0.5/1.0C_60_(OH)_24_	0.5/1.0
0.5/1.5C_60_	0.5/1.5	0.5/1.5C_60_(OH)_24_	0.5/1.5
0.5/2.0C_60_	0.5/2.0	0.5/2.0C_60_(OH)_24_	0.5/2.0
0.5/3.0C_60_	0.5/3.0	0.5/3.0C_60_(OH)_24_	0.5/3.0
0.5/5.0C_60_	0.5/5.0	0.5/5.0C_60_(OH)_24_	0.5/5.0
4.0/1.0C_60_	4.0/1.0	4.0/1.0C_60_(OH)_24_	4.0/1.0

## Data Availability

The original contributions presented in this study are included in the article. Further inquiries can be directed to the corresponding authors.

## Abbreviations

The following abbreviations are used in this manuscript:

DSC	Differential-scanning calorimetry
FTIR	Fourier-transform infrared (spectroscopy)
HPC	Hydroxypropyl cellulose
MWCNT	Multi-walled carbon nanotubes
PR	Piezoresistive (response)
TR	Thermoresistive (response)
SAXS	Small-angle X-ray scattering
SEM	Scanning electron microscopy
